# Modeling Adolescent Disposition Development: Age-Related Changes in Psychosocial Processes Correlated with Substance Use

**DOI:** 10.1007/s10935-024-00809-8

**Published:** 2024-09-21

**Authors:** William B. Hansen, Jared L. Hansen

**Affiliations:** 1 Prevention Strategies, LLC, Greensboro, USA; 2 Informatics, Decision-Enhancement and Analytic Sciences Center (IDEAS 2.0), VA Salt Lake City Health Care System, Salt Lake City, USA

**Keywords:** Modeling, Adolescents, Disposition, Intentions, Attitudes, Beliefs, Values

## Abstract

**Purpose:**

A model is proposed in which longitudinal changes in adolescents’ dispositions increase age-related risk for the onset of substance use.

**Method:**

Pooled surveys from 25 longitudinal studies were examined. Disposition was calculated from eight variables: use intentions; refusal intentions; attitudes; positive consequence beliefs; beliefs about negative consequences; descriptive peer normative beliefs; injunctive peer normative beliefs; and lifestyle incongruence. Substance use onset (past 30-day alcohol, cigarette, and marijuana use) was analyzed using participants’ just prior dispositional status and recent changes in their dispositions.

**Results:**

Disposition was highly correlated with each of the measured variables. The pattern of disposition changes as adolescents grow older, revealing that younger adolescents have more positive dispositions; whereas when they grew older, negative dispositions gradually emerged among a subset of adolescents. Analyses also revealed that dispositional status and recent changes in their dispositions were strong predictors of substance use onset.

**Implications.** Better understanding the development of dispositions will aid in designing effective interventions. Subordinate variables are amenable to intervention and are recommended as the primary focus of prevention programming. Because of the developmental trajectory of dispositions, multi-year interventions are highly recommended. Whenever possible, tailored approaches that take adolescents’ pre-intervention dispositions into account should be considered.

## Introduction

### Dispositions

From a psychological perspective, a disposition is a general trait that can be used to describe a person’s readiness and willingness to engage in a given behavior (Hyatt, Sleep, Weiss, and Miller, 2018). It is likely that individuals, especially adolescents, might find it difficult to describe their dispositions. Nonetheless, it is reasonable to assume that one’s disposition is a frame of reference that guides the propensity and willingness to engage in activities and respond to opportunities to behave in a given context or situation.

Early theorizing (Campbell, 1963) included dispositions along with attitudes. More recently, Mischel (2004) characterized dispositions as traits that persist across settings. There have been multiple studies that have conceptualized and attempted to measure adolescents’ dispositions. For example, dispositions have been examined in reference to adolescents’ smoking (Whalen et al., 2001), emotions and aggression (Susman et al., 1987), willingness to accede to peer pressure (Brown et al., 1986), antisocial behavior (Trentacosta et al., 2009), conduct disorder (Lahey et al., 2008), mindfulness (Kechter et al., 2019), and self-esteem (Anderson & Olnhausen, 1999). Willingness has also been distinguished from intentionality (Gibbons et al., 1998) and has been shown to be a predictor of adolescent alcohol consumption. While the basic concept of a disposition included in each of these studies reflects a similar framework, the measures available suggests that it can be approached in a variety of ways. This study examines adolescents’ dispositions to participate in or avoid substance use.

### Psychosocial Variables Related to Dispositions

By the 1960s, social psychologists had identified a number of variables for explaining observable behaviors. Primary among these was the role that attitudes played. The importance of attitudes was first introduced as a fundamental concept by early theorists (Allport, 1935; Heider, 1946, 1958; Krech & Crutchfield, 1948) who sought to characterize an individual’s motivation to behave. Since then, there have been a variety of ways in which attitudes have been conceptualized and measured (Briñol & Petty, 2012).

In its simplest formulation, attitudes may refer to liking or disliking, agreeing, or disagreeing, judging rightness or wrongness, or making some other affective analysis about a topic. Many who assess attitudes continue to think in this way, using scales in which survey participants are asked to make summary judgments. However, from early in the history of theory and research on attitudes, the concept has also been characterized as a complex phenomenon reflecting the accumulation of multiple components. For example, an early theory proposed that attitudes were based on three components: affect, beliefs, and behavior (Katz, 1960). In a similar vein that emphasized the multi-component nature of attitudes, Heider (1958) proposed that individuals actively strive to find balance among multiple components of attitudes.

Attitudes have been recognized to have a social perception component. For example, Festinger (1954) argued that people evaluate their attitudes in reference to the perceived attitudes of those around them. The nomenclature now refers to perceptions of others’ attitudes as either normative beliefs or injunctive norms. The companion to the perceived attitudes of others is the perception of others’ behaviors or descriptive social norms (Cialdini, 2007).

In addition to attitudes, there has been a concerted focus on values and the role that they play in people’s lives and the choices they make. In Rokeach’s theory (Rokeach, 1972, 1973), values are enduring beliefs that are intrinsic to individuals and define their preferences for desired end states of living. Individuals strive to achieve consonance between these preferences and their behaviors.

Ajzen & Fishbein (1977) theorized that attitude specificity (linking specific attitudes with behaviors versus linking general attitudes with behavior) was essential to understanding how attitudes function. In their early research (Ajzen & Fishbein, 1972) and in the theory of reasoned action (Fishbein & Ajzen, 1975, 1980), they examined a number of components of attitudes, including concepts related to the perceptions and judgments of the behavior of others and social norms, values, and intentions to behave.

Attitudes and social norms have not been the only psychosocial variables of interest. Intentions have also received extensive attention in the literature (McIntyre & Smith, 1989); however, it was not until this construct was included in research in the theories of Ajzen and Fishbein that it rose to prominence as a key variable in research. As a result, it became salient in the theory of reason action (Fishbein & Ajzen, 1975), the theory of planned behavior (Ajzen, 1985), and the reasoned action approach (Fishbein & Ajzen, 2010). Intentions and intentionality are found to be most predictive of behavior. The differences among these various theories include the addition or reconfiguration of a variety of variables. There is strong support for understanding the variety of psychosocial correlates of behavior that include intentions, attitudes, social norms, and values (Hagger et al., 2022).

A similar set of variables (excluding intentions) was proposed by Jessor (Jessor, 1991; Jessor & Jessor, 1977). In problem behavior theory, the personality system include a patterned and interrelated set of enduring, socio-cognitive variables that include values, expectations, beliefs, attitudes, and orientations toward self and society. Additional models have since been developed (Flay, Snyder, & Petraitis, 2009; Montano & Kasprzyk, 2015) that further include a much wider array of variables.

In light of this prior empirical research, intentions, attitudes, injunctive and descriptive norms, beliefs about the consequences of behavior, and the perceived value of behavior have all been viewed as central to predicting behavior (Sheppard et al., 1988). This paper addresses these psychosocial variables from a developmental perspective and explores the degree to which the development of dispositions influence substance use onset.

## Method

### Source of Data

Data for the analyses presented here were from the Hansen et al., 2022 dataset (publicly available at: 10.5281/zenodo.5256140). This dataset includes surveys accumulated from 25 studies that contributed control group data to the database. Treatment group data, if it existed, were excluded from dataset creation.

Data included in this dataset come from 80,532 participants who, collectively, completed 344,429 surveys. All studies included at least two waves of data and several included as many as seven waves. One study included twenty-six waves of data. Each study included in the pooled dataset included information about participants’ gender and age (see Table 1) and participants’ ethnicity and race (see Table 2). Each project collected data in an analogous manner with participants tracked across time using a de-identified ID number. The exact timing of each wave of data collection varied but typically involved lags between waves of three to six months with some projects administering surveys annually.

**Table 1:** Number of cases, surveys, waves, gender, and age composition of studies

					Age		
Study	Participants	Surveys	Waves	% Female	Minimum	Maximum	Mean
1	3,276	22,060	6	50.80%	10.00	18.52	13.32
2	2,340	9,654	5	53.80%	10.06	18.47	13.52
3	3,103	17,851	5	52.80%	10.00	18.25	12.84
4	2,984	12,658	5	52.70%	10.12	18.95	14.66
5	649	3,092	6	54.20%	10.00	15.00	12.20
6	2,274	6,060	3	53.00%	11.00	16.00	12.85
7	4,039	11,545	3	55.50%	10.50	18.5	13.64
8	2,912	6,437	3	53.50%	13.00	18.00	14.89
9	2,503	7,349	3	51.30%	10.00	19.00	12.59
10	8,747	23,474	4	62.20%	17.52	20.00	18.94
11	6,736	47,120	7	49.60%	10.00	19.98	14.72
12	562	2,457	6	55.30%	13.87	20.00	16.63
13	2,651	9,274	4	48.00%	11.91	17.91	14.48
14	5,418	21,840	6	52.10%	10.01	18.75	12.46
15	3,358	14,554	4	49.80%	10.00	20.00	14.20
16	1,180	4,355	4	47.20%	11.00	17.00	13.35
17	6,467	10,807	2	50.40%	12.00	20.00	15.23
18	219	1,758	8	46.40%	10.02	19.80	13.80
19	446	3,070	4	48.60%	10.00	18.00	12.75
20	761	1,951	3	50.00%	14.50	20.00	17.02
21	2,471	6,411	2	53.50%	10.76	19.93	14.96
22	389	1,156	3	100.00%	10.00	16.00	14.26
23	331	860	3	60.10%	11.66	19.29	14.49
24	15,693	85,854	7	56.50%	10.00	20.00	14.08
25	1,023	12,782	26	55.40%	10.60	20.00	15.67
	80,532	344,429	5.28	54.50%			14.30

**Table 2:** Ethnicity and race composition of studies*

Study	Black %	White %	Native American %	Hispanic %	Asian %	Pacific Islander %	Other/ Multi %
1	4.0	53.3		29.6	13.0		
2	2.7	35.1		47.2	5.9		9.1
3	3.9	44.2		29.1	4.4		18.4
4	3.1	46.8		33.6	15.0		1.5
5	84.	0.0	0.8	0.3	0.0		14.6
6	24.	67.5	1.2	1.7	2.4	0.2	2.1
8	15.9	70.1	0.4	5.4	2.5	0.5	5.0
9	12.2	44.6	1.4	26.7	8.5	0.9	5.6
10	19.5	50.5	0.5	6.0	16.2	0.6	6.6
11	37.2	52.5	1.2	4.0	0.5		4.6
12	80.8	16.6					2.6
13	1.7	90.9	1.2	1.4	0.8	1.2	2.7
14	37.8	22.6	3.2	19.3	7.2	3.5	6.3
16	1.4	88.8		2.1	0.4		7.1
17	2.7	89.7	0.7	1.0	1.4		4.4
18	31.5	45.2	3.9		19.4		
19	11.2	85.8		3.0			
20	85.7	8.1	5.7				0.5
21	16.3	47.9	1.2	21.7	2.8		10.1
22	19.3	52.2	0.3	6.7	2.6		19.0
23				100.0			
24	9.4	51.0	4.8	21.9	4.3		8.7
25	3.9	72.8	1.7	12.5	2.9	0.4	5.9
Overall	17.0	51.9	2.0	16.6	5.4	0.4	6.6

*Ethnicity/race information was provided for 87.7% of surveys (N = 302,194)

Insert Tables 1 and 2 here.

### Measures

The pooled data included past 30 day dichotomous (yes/no) self-reports of drinking alcohol, smoking cigarettes, and using marijuana. Relevant to the psychosocial research reviewed above, eight psychosocial variables were selected for inclusion in planned analyses. Variables were selected based on the magnitude of correlations observed in Hansen et al. (2020). These included (1) intention to use or not use substances, (2) intention to refuse substance use offers, (3) attitudes toward substance use, (4) positive beliefs about substance use, (5) negative beliefs about substance use, (6) descriptive peer norms, (7) injunctive peer norms, and (8) perceived lifestyle incongruence with substance use. In only a few instances did projects use the same exact items. Nonetheless, in most cases, classification was unambiguous once conceptual criteria were defined. Sample items reflective of these eight categories follow.

- I have made a firm decision to not smoke cigarettes. (use intentions)
- If I was offered the chance to vape, I would take it. (refusal intentions)
- It is not okay to use marijuana. (attitudes)
- Drinking will help me be popular. (positive beliefs)
- Smoking cigarettes would harm my health. (negative beliefs)
- My friends think that using marijuana is stupid. (injunctive normative beliefs)
- How many students your age get drunk every month? (descriptive normative beliefs)
- Getting drunk every now and then is how I want to live my life. (lifestyle incongruence)

Average Cronbach alpha coefficients for each of the eight dispositional variables ranged from 0.777 to 0.866, with a mean of 0.818 (see Table 3). All psychosocial variables were scored such that greater values represented more theoretically and socially desirable outcomes. Scores for each item were normalized with theoretically worst scores recoded to be 0 (zero) and best scores recoded to be 10 (ten) with intermediate responses equally distributed between the extremes. For example, if the original scale had responses of 1, 2, 3, 4, and 5 with a score of 5 being most desirable, normalization resulted in scale values of 0.0, 2.5, 5.0, 7.5, and 10.0. When most desirable outcomes were the lowest score, as was typically the case with measures of descriptive normative beliefs, scale values were reversed. Scales were created by averaging relevant items. This allowed all items and scales to share the same 0-to-10 range.

**Table 3:** Numbers of studies contributing cases and average Cronbach alpha coefficients

	Studies	Alpha	Std Dev
Attitude	12	.835	0.104
Beliefs (Negative)	17	.781	0.126
Beliefs (Positive)	17	.826	0.109
Intention to Avoid Use	18	.777	0.109
Intention to Refuse Offers	7	.866	0.068
Lifestyle Incongruence	4	.784	0.090
Peer Norms (Descriptive)	24	.833	0.070
Peer Norms (Injunctive)	15	.855	0.083

Insert Table 3 here.

### Methodological Innovation

This study used pooled raw data from multiple studies and, as such, represents a novel approach to synthesizing and analyzing data. Unlike meta-analysis (Becker et al., 2020) and systematic review (Higgins, et al., 2019) that combine results from published studies, this study was able to use multiple projects’ survey responses. It also differs from integrative data analysis (Curran & Hussong, 2009) in that rather than standardizing variables (typically with means of zero and standard deviations of one) using raw data, data in this study normalized data as described above.

### Analysis Plan

Primary analyses consisted of binary correlations using survey psychosocial and substance use variables from available surveys irrespective of the wave during which the data were collected. Analyses were completed using SPSS version 28.

Lagged correlation coefficients for each of the eight variables as well as a summary disposition variable that was the mean of each of the individual eight variables were calculated. Lagged correlations were based on data from subsequent waves. Therefore, wave 1 to wave 2 correlations contributed to T → T + 1, as did correlations for 2 to 3, 3 to 4, 4 to 5, 5 to 6, and 6 to 7. Similarly, T → T + 2 correlations included wave 1 to wave 3, 2 to 4, 3 to 5, 4 to 6, and 5 to 7, and so forth for T → T + 3, T → T + 4, T → T + 5, and T → T + 6.

Disposition was calculated by averaging available measures from each study. In all, disposition measures were available for 304,610 surveys. Using these surveys, age-specific scores for adolescents at the 10th, 20th, 30th, 40th, 50th, 60th, 70th, 80th, and 90th percentiles for ages 120 months (10 years) to 240 months (20 years) were calculated. Data for each percentile group smoothed across ages using a quadratic function.

An analysis was conducted to examine the individual dispositional development profiles of participants who had scores across six waves of data. The pool of participants who met this qualification limited analyses to surveys from 8,107 participants. Analysis was conducted using R version 4.4.1. Analyses were completed using a linear mixed effects model with an AR1 correlation structure and random effects for wave and ID to estimate the fixed effects of age on disposition. A second model was analyzed to account for the interaction between age and initial disposition that excluded wave 1 from the regression. Age and initial disposition were scaled to facilitate model convergence, leaving us to interpret the results in the context of a one standard deviation change in age and initial disposition (1.52 years and 1.85 units, respectively).

To assess the potential for dispositions to serve as predictors of the onset of drinking alcohol, smoking cigarettes, and using marijuana, logistic regression analyses were performed using SPSS version 28. Analyses evaluated three predictors: the prior wave disposition score, changes in a participant’s disposition score between the just prior wave and the current wave, and the participant’s age at the current wave. The dependent variables consisted of participant’s use status at the current wave: either continuing non-use (abstaining) or reporting use (initiating use). Data were available for predicting onset for waves 2 through 6. Only prior wave abstainers were included in the analysis of each wave’s data.

## Results

### Psychosocial Correlates

Because studies included differing numbers of psychosocial variables, confirmatory factor analysis was not possible. Instead, correlations among all variables were calculated. The strongest correlations were between the disposition variable and each of the measured psychosocial variables. All but two correlations were above 0.700.

Insert Table 4 here.

**Table 4:** Bivariate correlations between each measured variable and disposition

		Disposition	Attitudes	Beliefs (Negative)	Beliefs (Positive)	Intention to Refuse	Intention to Avoid Use	Lifestyle Incongruence	Peer Norms (Descriptive)
Attitude	*r*	.785							
	Cases	161,714							
Beliefs (Negative)	r	.667	.457						
	Cases	201,388	119,915						
Beliefs (Positive)	*r*	.731	.443	.279					
	Cases	199,770	121,970	169,052					
Intention to Avoid Use	*r*	.781	.606	.419	.491				
	Cases	209,677	144,002	134,651	138,634				
Intention to Refuse Offers	*r*	.729	.638	.194	.471	.648			
	Cases	40,880	4,558	31,004	30,334	18,891			
Lifestyle Incongruence	*r*	.757	–	.626	.543	.550	.502		
	Cases	32,585	–	21,160	14,780	6,335	31,147		
Peer Norms (Descriptive)	*r*	.699	.324	.188	.346	.302	.447	.301	
	Cases	254,417	159,373	161,831	177,437	39,394	201,116	32,450	
Peer Norms (Injunctive)	*r*	.791	.551	.240	.417	.374	.466	.388	.537
	Cases	203,641	136,361	133,427	142,691	27,416	180,435	30,764	203,129

### Lagged Correlations

Table 5 presents the results of the lagged correlation analysis. The duration of the time that elapsed between the various waves of data collection in the 25 studies varied, and thus the periods cannot be specified in terms of lags of weeks, months, or years.

**Table 5:** Averaged lagged correlations across time for measured variables and disposition

		T → T + 1	T → T + 2	T → T + 3	T → T + 4	T → T + 5	T → T + 6
Disposition	*r*	.647	.532	.441	.373	.324	.352
	Cases	176,265	122,186	77,803	48,026	24,862	10,136
Attitudes	*r*	.556	.442	.361	.290	.236	.209
	Cases	105,172	73,777	49,521	31,349	17,583	7,778
Beliefs (Negative)	*r*	.6	.532	.479	.423	.381	.357
	Cases	113,370	79,899	47,489	31,941	19,809	9,507
Beliefs (Positive)	*r*	.514	.396	.316	.253	.199	.163
	Cases	106,439	74,613	47,651	33,042	17,662	7,763
Intention to avoid use	*-r*	.622	.509	.437	.368	.333	.306
	Cases	129,067	90,071	57,413	37,150	19,031	8,002
Intention to refuse	*r*	.458	.271	.095	.023	.031	–
	Cases	17,961	12,437	5,034	2,614	1,370	–
Lifestyle incongruence	*r*	.536	.493	.405	.385	.326	.334
	Cases	15,268	8,508	2,749	1,949	1,178	347
Peer Norms (Descriptive)	*r*	.509	.467	.399	.297	.259	.244
	Cases	146,118	101,797	64,064	40,614	19,535	8,215
Peer norms (Injunctive)	*r*	.526	.419	.354	.271	.206	.211
	Cases	117,577	82,423	53,398	33,539	18,287	8,155

Insert Table 5 here.

As the interval between initial measurement and subsequent measurement increases, the magnitude of the correlation decreases. One would expect this given the numerous changes that occur during adolescent development. Three variables, overall disposition, adolescents’ intentions regarding use, and negative beliefs about consequences, revealed lagged correlations that were noticeably stronger than the remaining variables for all calculated lags.

### Correlations with Substance Use

As would be expected from prior research, each of the eight variables were strongly correlated with past 30 day substance use. Intention to avoid use was the single strongest correlate with 30-day use followed by attitudes toward use. Beliefs about negative consequences was consistently the weakest correlate. While not as strong as intentions to avoid use, disposition was nonetheless a strong correlate.

Insert Table 6 here.

**Table 6:** Bivariate correlations between self-reported 30 day use and measured variables and disposition

	Alcohol		Cigarettes		Marijuana	
	*r*	Cases	*r*	Cases	*r*	Cases
Disposition	− 0.519	230,844	− 0.499	251,992	− 0.453	221,955
Attitude	− 0.427	136,071	− 0.437	157,767	− 0.378	139,851
Beliefs (Negative)	− 0.249	143,096	− 0.236	163,519	− 0.218	142,277
Beliefs (Positive)	− 0.398	155,402	− 0.329	177,487	− 0.294	157,799
Intention to avoid use	− 0.522	187,335	− 0.555	200,703	− 0.495	190,134
Intention to refuse offers	− 0.384	40,193	− 0.393	40,428	-0.396	40,398
Lifestyle Incongruence	− 0.318	31,458	-0.306	31,716	-0.399	26,211
Peer Norms (Descriptive)	− 0.377	215,460	− 0.328	238,264	− 0.327	215,332
Peer Norms (Injunctive)	− 0.370	190,419	− 0.328	194,825	− 0.297	186,844

### Adolescent Disposition Development

**Fig. 1:**
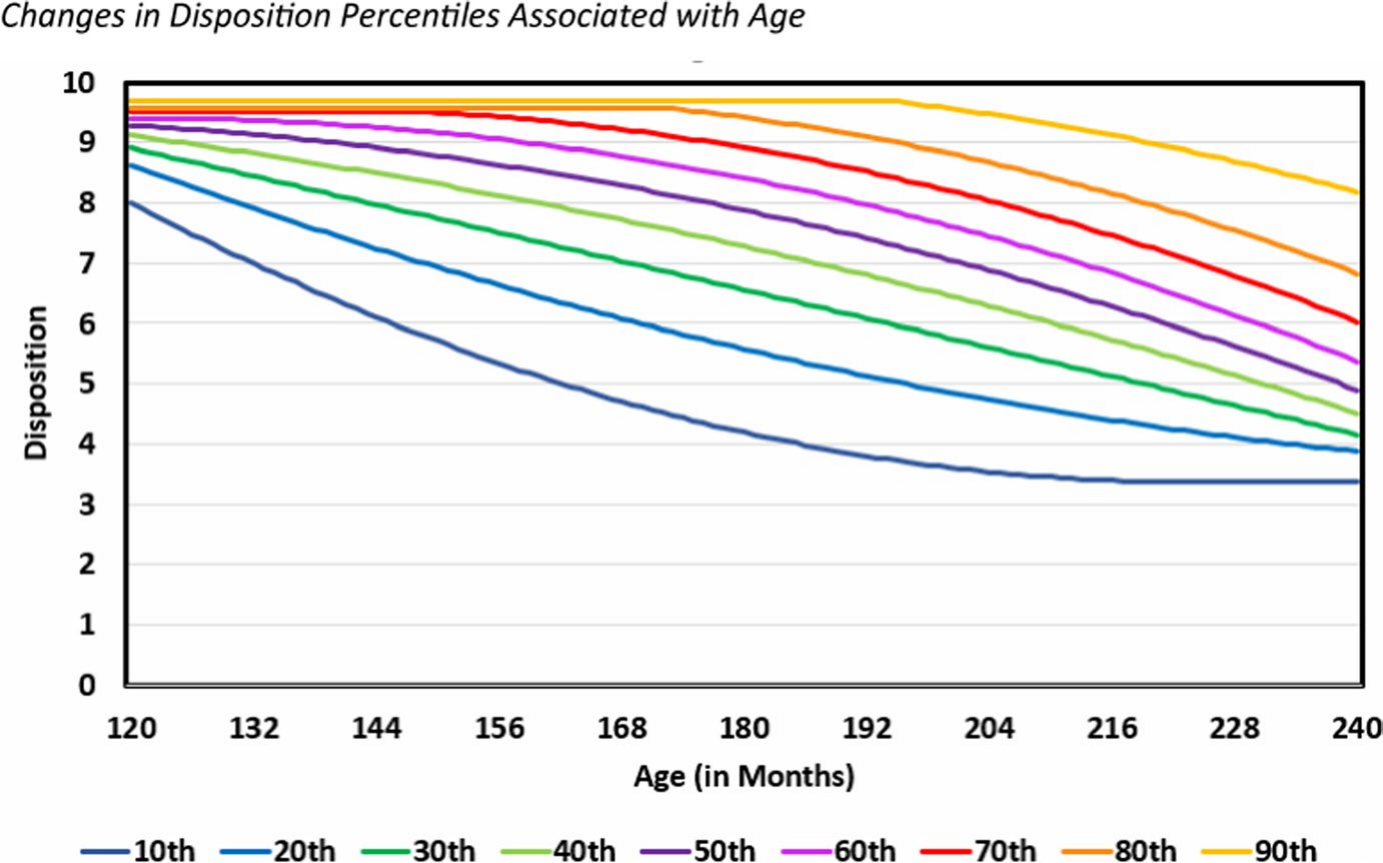
Changes in disposition percentiles associated with age

Generally speaking, across adolescence, dispositions develop in a manner that reflects a trend for increasing diversity (see Fig. 1). For example, the difference in disposition scores comparing the 10th to the 90th percentiles at age 120 months (10 years) is 1.7 whereas the difference between the 10th and 90th percentiles at age 198 months (16 years 6 months) is 6.0.

Insert Fig. 1 here.

As can be deduced from the lines presented in Fig. 1, the difference that emerges over time is primarily due to changes in lower percentiles which erode rapidly with age, and hence, hypothetically reflect an accelerating willingness to participate in substance use. It should be noted that scores in the 10th through 60th percentiles gradually become lower immediately after age 120 months (age 10). It is not until after 198 months (16 years 6 months) that the 90th percentile disposition scores start to decline, and then only slightly.

Only the summary disposition variable is presented in Fig. 1. However, because it is an amalgam of the eight measured variables, it should be noted that each of the contributing variables (use and refusal intentions, attitudes, positive and negative beliefs, descriptive and injunctive normative beliefs, and lifestyle incongruence) all share a similar pattern of development across adolescence.

Dispositional development across waves was examined using data from those who were present at six consecutive waves of data collection. After fitting longitudinal data using the linear mixed effects model (Diggle et al., 2002), incrementing age by one standard deviation (1.52 years) was associated with a 0.53 decrease in disposition (SE = 0.01, *p* < 0.001). The second model adjusted for age, initial disposition, and the interaction between them. It resulted in a similar effect of age (*β* =  − 0.53, SE = 0.01, *p* < 0.001), a protective effect of incrementing initial disposition by one standard deviation (1.85 units) (*β* = 0.87, SE = 0.02, *p* < 0.001), and a decrease in disposition associated with the interaction between initial disposition and age (*β* =  − 0.16, SE = 0.01, *p* < 0.001).

### Predicting Substance Use Onset

From wave 1 to wave 6, 42.1% of abstainers initiated alcohol use. Similarly, 18.3% of survey participants who were abstinent from smoking cigarettes began smoking by wave 6. Slightly fewer (16.4%) reported transitioning from being abstinent at wave 1 to using marijuana at wave 6.

Table 7 presents results of the logistic regression analyses. For all three behaviors and all waves of data, two variables (prior wave disposition and change in disposition from the just prior wave) were significant predictors of participants making a transition from abstinence to the initiation of substance use. Participants’ age was not a predictor of making the transition to use at any wave.

**Table 7:** Prediction of transitioning from no use to use, results of logistic regression

	Wave 2					Wave 3					Wave 4					Wave 5					Wave 6			
	OR		95% CI			OR		95% CI			OR		95% CI			OR		95% CI			OR		95% CI	
Alcohol																								
Prior disposition	0.48	†	0.44	0.52		0.38	†	0.35	0.41		0.41	†	0.37	0.44		0.40	†	0.37	0.43		0.42	†	0.39	0.46
Change in disposition	0.39	†	0.36	0.43		0.36	†	0.33	0.39		0.38	†	0.35	0.42		0.41	†	0.38	0.44		0.47	†	0.43	0.51
Age at the current wave	1.30		1.10	1.54		1.14		0.99	1.30		1.16		0.99	1.36		0.87		0.75	1.01		1.17		1.00	1.37
Cigarettes																								
Prior disposition	0.37	†	0.33	0.41		0.40	†	0.36	0.45		0.38	†	0.34	0.42		0.38	†	0.34	0.41		0.40	†	0.37	0.44
Change in disposition	0.38	†	0.34	0.42		0.35	†	0.32	0.39		0.41	†	0.37	0.45		0.34	†	0.31	0.37		0.41	†	0.38	0.45
Age at the current wave	1.15		0.92	1.44		1.26		1.05	1.50		1.23		1.02	1.48		1.00		0.83	1.20		1.05		0.87	1.25
Marijuana																								
Prior disposition	0.33	†	0.29	0.39		0.34	†	0.30	0.39		0.37	†	0.33	0.42		0.36	†	0.33	0.40		0.35	†	0.31	0.38
Change in disposition	0.32	†	0.28	0.38		0.34	†	0.30	0.37		0.38	†	0.34	0.42		0.36	†	0.33	0.39		0.33	†	0.29	0.36
Age at the current wave	1.21		0.88	1.65		1.28		1.04	1.57		1.12		0.90	1.40		0.96		0.79	1.17		0.86		0.70	1.07

^†^*p* < .001

Insert Table 7 here.

To aid in interpreting these findings, prior wave disposition and change in disposition scores are presented in Table 8. Comparing those who initiated use with those who remained abstinent, prior wave disposition scores were lower for those who initiated use. Across all waves, continuing abstainers from alcohol average prior wave disposition score was 8.07 versus 7.25 for initiators, a difference of 0.82. Similarly, even though alcohol abstainers saw a decrease in the disposition scores from wave-to-wave (− 0.23 on average), wave-to-wave decreases for alcohol initiators were larger (− 1.12 on average).

**Table 8:** Average dispositions and changes in dispositions of abstainers and substance use Initiators

	Wave 2			Wave 3			Wave 4			Wave 5			Wave 6	
	Abs	Init		Abs	Init		Abs	Init		Abs	Init		Abs	Init
Alcohol														
Prior Wave Disposition	8.33	7.39		8.25	7.50		8.37	7.59		7.67	6.86		7.73	6.89
Change in Disposition	− 0.22	− 1.27		0.02	− 1.41		− 0.89	− 2.02		− 0.11	− 1.14		0.07	0.24
Cigarettes														
Prior Wave Disposition	8.24	6.98		8.05	7.11		8.04	6.72		7.21	6.24		7.17	6.09
Change in Disposition	− 0.25	− 1.40		− 0.07	− 2.22		− 0.92	− 2.04		− 0.14	− 1.67		0.11	0.35
Marijuana														
Prior Wave Disposition	8.17	6.71		7.96	6.85		7.93	6.63		7.04	5.94		7.07	5.90
Change in Disposition	− 0.26	− 1.69		− 0.09	− 2.36		− 0.99	− 2.29		− 0.07	− 1.42		0.11	− 1.33

Abs = Abstainers, Init = Initiators

Insert Table 8 here.

Comparable results were observed for the differences between cigarette and marijuana use abstainers and initiators. Cigarette abstainers had average prior wave dispositions of 7.74 whereas those who initiated cigarette smoking had prior wave dispositions of 6.63, a difference of 1.12. On average, the decreases in wave-to-wave disposition scores for cigarette abstainers was 0.25 whereas initiators decreased an average of 1.40. Comparing marijuana abstainers and initiators’ scores yielded similar results. Abstainers’ average prior wave disposition scores were 7.64 compared to 6.41 for marijuana initiators. Wave-to-wave decreases in scores for abstainers was 0.26 compared to 1.82 for marijuana initiators.

## Discussion

There are several reasons why creating a disposition variable is justified. Aggregating intentions, attitudes, beliefs about consequences, normative beliefs, and lifestyle incongruence into a single latent variable made analysis both manageable and interpretable. This reduced redundancy and eliminated potential multicollinearity. The resulting disposition variable proved to be strongly correlated with each of the subordinate variables, suggesting that it improved reliability and precision. An individual’s disposition and changes in disposition over time was conceptually meaningful; it captured an essential characteristic that proved useful in predicting the onset of substance use. Finally, having a single variable allowed results to be easily interpreted. The wave-to-wave longitudinal correlations and the autoregressive nature of dispositions provide a means for thinking about developmental issues.

Dispositions reflect a propensity and willingness to behave. The variables that contribute to one’s disposition are the same or similar to variables noted in the Ajzen and Fishbein theories (Ajzen, 1985; Fishbein & Ajzen, 1980, 2010) and Jessor’s problem behavior theory (Jessor, 1991; Jessor & Jessor, 1977). These include intentions, attitudes, beliefs about social norms, beliefs about the consequences associated with behaviors, and perceived congruence or incongruence between behavior and a desired lifestyle.

Four points are worthy of note. First, as the lagged correlations in Table 5 suggest, dispositions are more or less consistent across time, at least in the short term. Over time, an adolescent’s disposition is likely to be more consistent than any of its contributing measures and thus reflects a meaningful psychosocial construct. This suggests that an individual’s disposition reflects a stable characteristic.

Second, as seen in Table 6, the psychosocial elements that are indicators of disposition are highly correlated with substance use. Adolescents’ self-reports about behavior are always suspect in that they may have a social desirability bias to under-report use or may simply have poor recall. Nonetheless, finding strong correlations between prior dispositions, wave-to-wave changes in dispositions, and behaviors points to possible real relationships.

Third, even though lagged correlations are strong, especially in the short run, the data demonstrate a great deal of individual variability. As noted in the analysis of participants’ longitudinal data, there may be a great deal of time-to-time variability in an individual’s disposition. In all repeated measurement situations, one expects regression to the mean. In the case of dispositions, the overall mean changes at the same time that variability in the population increases. The development of an individual’s disposition across time may thus be complex. Nonetheless, without an intervention that would alter one’s trajectory, an individual’s age-specific disposition can be expected to track with others who share similar dispositions.

Fourth, the increasing spread in percentiles in Fig. 1 reflects the increasing diversity in dispositions that are typical of the changes that one sees in adolescents as they transition through this decade of life. There are individuals (characterized as those in the 70th through 90th percentiles) who retain a disposition to avoid risky or deviant behaviors through their middle adolescent years. Median cases (portrayed as the 50th percentile) gradually and increasingly change in their dispositions that reflect increases in their risk for using substances, particularly after age 15. On the other hand, changes in the lower percentiles (10th through 40th) reflect dispositions that may place them at risk at young ages.

Causality is a fundamental issue with any model of behavior. Causation cannot be confirmed by means of correlation. Nonetheless, the as adolescents aged from wave-to-wave, the prior wave’s disposition and the prior-to-current wave change in disposition support thinking that changes in dispositions precede the onset of substance use. Future research may explore alternative explanations of these findings.

### Implications

These psychosocial variables (intentions, attitudes, normative beliefs, beliefs about consequences, and lifestyle incongruence) have been included in numerous research studies and have been found to be predictive of a variety of adolescent behaviors including cigarette smoking (Grube et al., 1986; Morgan & Grube, 1994), alcohol consumption (Caputo, 2020; Jessor, 1991; Morgan & Grube, 1997; Zhao, et al., 2020), and marijuana use (Bearden & Woodside, 1978a, 1978b; Korn et al., 2021).

While one’s disposition may be a social psychological construct that determines an adolescent’s risk for engaging in deviant behaviors, there is no implication that dispositions exist in a vacuum. Indeed, the social influences of peers (Duan et al., 2009; Stacy et al., 1992; Unger et al., 2001), parents and families (Deković et al., 2003; Goulter et al., 2020; Hansen et al., 1987; LoBraico et al., 2020), schools (Barker, Brown, Pitpitan, Shakya, & Raj, 2023; Hansen, Beamon, Orsini, & Wyrick, 2023), and communities (Jessor & Jessor, 1977; Mennis & Mason, 2016; Treno, Grube, & Martin, 2003; Tunstall, Shortt, Pearce, Mitchell, & Richardson, 2016) all play a role in fostering one’s disposition toward a wide variety of specific topics as well as one’s general disposition to engage in deviant or conventional behaviors.

The implications for intervention are clarified by understanding the centrality of disposition development. It may not be possible to address dispositions directly. However, as program developers create interventions, adopting a dispositional framework will help guide the kinds of structures and activities that may help ensure their success. There are numerous prevention programs that focus on one or more of the subordinate constructs (Botvin et al., 1992; Hansen & Graham, 1991; McNeal et al., 2004). One of the key features of disposition-focused interventions is the bringing of these constructs from a preconscious level to one in which consciousness and cognitive attentiveness are promoted.

These results support extending interventions in two ways. First, age-specific target group percentiles may be used as a marker of risk. The implication is that interventions should target as many of the subordinate variables as possible. Interventions should then seek to maintain (in the case of positive dispositions) or improve (in the case of worse-than-average dispositions) the constituent constructs.

Interventions may be tailored to the risk status of the target group. For example, for at-risk groups, interventions should bolster intentions to avoid the riskiest behaviors and promote intentions to refuse invitations to participate in these. Such interventions may utilize strategies for correcting erroneous normative beliefs and promoting conscious awareness of desired futures. Strategies that encourage and reward the reconsideration of intentions may yield positive outcomes.

Prevention programs delivered to lower-risk classes of students can assume the role of reinforcing positive intentions, attitudes, beliefs about consequences, low-use social norms, and perceptions of incongruence between substance use and desired lifestyles. For each specifically targeted mediator, interventions should strengthen already existing positive dispositions and ameliorate dispositions that are less than ideal. Moreover, program facilitators may benefit from an understanding of students’ dispositions. This may have an impact on how their day-to-day interactions can be adapted to maintain positive dispositions or promote positive changes in their disposition status.

Second, because the developmental trajectories portrayed in Fig. 1 span much of early adolescence (through age 16), there need to be multiple and repeated opportunities for intervention. It is during these years that adolescents’ dispositions diverge, reflecting increased risk. Year-over-year repeated interventions may induce a positive change in these trajectories (Dykstra et al., 2023).

### Limitations

Pooling data from multiple studies potentially allows for more robust conclusions to be drawn and for greater generalizability across different populations and settings. At the same time, there is no guarantee that these outcomes have been achieved. It should be noted that the datasets included in these analyses spanned over 40 years of data collection, some dating back to the 1980s. In the interim, data collection methods may have changed in either dramatic or subtle ways that may influence observed outcomes. More recent data may yield different results.

The data actually procured may not necessarily reflect to potential pool of data that may exist. Approximately 40 researchers had been approached about including their raw data in the project. Many simply failed to respond. Several refused outright. Several who initially agreed to share subsequently either could not locate their data or failed to follow through. All data actually received were from U.S. studies, limiting the generalizability to other populations.

## Conflict of interest

The author has no conflicts of interest.

## Ethical Approval

Analyses involved only the use of existing publicly available data and was exempt based on 45 CFR part 46 subpart D exemption 4.

### Informed Consent

This study involved only secondary analysis of de-identified data.
